# Impact of Patient Resilience on Outcomes of Open Brostrom-Gould Lateral Ligament Repair

**DOI:** 10.5435/JAAOSGlobal-D-21-00103

**Published:** 2021-11-18

**Authors:** Nicholas A. Andrews, Aseel Dib, Timothy W. Torrez, Whitt M. Harrelson, Tanvee Sinha, Vyshnavi Rallapalle, Abhinav Agarwal, Ashish Shah

**Affiliations:** From the Department of Orthopaedic Surgery, University of Alabama at Birmingham; Birmingham, AL.

## Abstract

**Introduction:**

Little is known about the factors affecting the intermediate outcomes of the Brostrom-Gould repair as measured by new patient-reported outcome instruments and the impact of patient resilience on postoperative outcomes. This is the first study to investigate the impact of resilience on the outcomes of lateral ligament repair.

**Methods:**

Retrospectively, 173 patients undergoing Brostrom-Gould at single institution from January 2013 to June 2020 were identified. Patient characteristics, participation in athletic activities, surgical variables, and complications were recorded. Patient-Reported Outcome Measurement Information System (PROMIS) Pain Interference v1.1 (PI), Physical Function v1.2 (PF), and the Foot Ankle Ability Measure (FAAM) were collected. The Brief Resilience Scale was used to quantify resilience. A linear regression model was constructed to evaluate the independent effect of resilience on each PROMIS and FAAM outcome instrument. Variables were included in the regression model based on an a priori significance threshold of *P* <0.05 in bivariate analysis.

**Results:**

Resilience's independent effect on outcome measures was as follows: PROMIS PF (unstandardized β 8.2, 95% confidence interval [CI] 3.9 to 12.6), PROMIS PI (unstandardized β −4.8, 95% CI −7.9 to −1.7), FAAM Activities of Daily Living (unstandardized β 16.6, 95% CI 8.7 to 24.6), and FAAM Sports (unstandardized β 28.4, 95% CI 15.9 to 40.9). Preoperative participation in athletic activities also had a positive independent effect on multiple outcome metrics including PROMIS PF (unstandardized β 9.4, 95% CI 2.8 to 16.0), PROMIS PI (unstandardized β −5.3, 95% CI −10.0 to −0.582), and FAAM Sport scores (unstandardized β 34.4, 95% CI 15.4 to 53.4).

**Conclusions:**

Resilience and patient participation in athletic activities are independent predictors of improved postoperative functional outcomes as measured by PROMIS and FAAM instruments at intermediate term follow-up. Resilient patients and athletes reported markedly higher PF and less pain burden postoperatively. Preoperative quantification of resilience could enable improved prognostication of patients undergoing lateral ligament repair of the ankle.

Resilience can be defined as the “ability to recover from or adjust easily to misfortune or change.”^1^ Resilience in health care is the ability for a patient to adapt to stressful and traumatic situations to uphold or return to a normal state of functioning. Increased resilience has been found to affect the ability of patients with chronic conditions such as multiple sclerosis, spinal cord injuries, and multiple myeloma to cope with disability and pain.^1–3^ Given these properties, research has begun to emerge investigating the impact of resilience on patients' recovery from orthopaedic surgery.^4,5,6,7^ Otlans et al,^7^ in a recent review, summarized the growing evidence for the use of resilience to prognosticate clinical outcomes in orthopaedics. Importantly, currently no studies exist in literature describing the impact of resilience on the outcomes of foot and ankle surgery.^7^

Chronic ankle instability (CAI) is diagnosed if patients continue to experience symptoms and decreased function 6 months after the original lateral ankle injury.^8^ CAI can alter the biomechanics of the ankle joint with progression to cartilaginous damage and secondary osteoarthritis. Intra-articular and extra-articular ankle pathologies are associated with CAI including peroneal tenosynovitis, peroneal tears, pes varus alignment, and osteochondral lesions of the talus or tibia.^9,10^ Surgical treatment is commonly performed via an anatomic repair using the Brostrom-Gould technique. This techniques uses remnants of the anterior talofibular ligament to directly repair the injured ligament and reinforcement of the inferior extensor retinaculum to improve mechanical strength.^11^ The Brostrom-Gould technique is considered the benchmark in treatment of chronic lateral ankle instability with excellent short- and long-term outcomes.^12,13,14,15,16,17,18^

Patient-reported outcome instruments have become increasingly popular in orthopaedic surgery as a tool to obtain a holistic view of patients' postsurgical levels of function and pain. As such, the design of patient-reported outcome instruments have increased in complexity over time with new generations of instruments featuring improved psychometric and clinical properties. The Patient-Reported Outcome Measurement Information System (PROMIS) system is part of the next generation of outcome instruments and relies on a computerized adaptive algorithm to increased accuracy and decrease the survey burden for patients.^19^ Multiple PROMIS domains have been validated for use in the foot and ankle population, but few studies to date have used this new system to evaluate the outcomes of lateral ligament repair.^20^ In contrast to PROMIS scores, the older validated Foot and Ankle Ability Measure (FAAM) has been more widely used to examine the surgical results of patients with CAI.^15,20,21,22,23,24,25^

Many studies have examined potential risk factors for the recurrence of instability and poor prognosis after Brostrom-Gould repair. Osteochondral defects, generalized ligamentous laxity, obesity, and the degree of instability have all been found to be possible predictors of poor outcomes.^16,26,27^ However, most of these previous studies have used composite outcome metrics and a variety of outcome scores including scoring instruments with unacceptable psychometric characteristics.^17^ In this study, we seek to investigate the role of patient resilience and other potential factors affecting the intermediate term outcomes of the modified Brostrom-Gould repair as measured by the FAAM and PROMIS instruments. To our knowledge, this is the first study to investigate the impact of resilience on the outcomes of lateral ligament repair.

## Methods

After institutional review board approval, all patients who underwent open modified Brostrom-Gould lateral ligament repair were identified using the Current Procedural Terminology code 27698 from January 2013 to June 2020 at a single academic institute. Only patients undergoing modified Brostrom-Gould repair for CAI, defined as the presence of symptoms for at least 6 months, were included in this study. Patients who received suture tape augmented repairs were also excluded. After these exclusions, a total of 173 patients met the inclusion criteria.

Patient medical records were examined to collect patient demographics and comorbidities. Patient participation in athletic activities was also noted and later confirmed in a telephone survey. Information related to copathologies commonly seen with CAI including the presence of osteochondral defects, peroneal tendinopathy, deltoid ligament injury, varus talar tilt, anterior ankle impingement, posterior ankle impingement, and ankle osteoarthritis in preoperative radiographs were recorded. Procedural information including the use of ankle arthroscopy, calcaneal osteotomy, peroneal repairs or augmentation, and peroneal longus to brevis transfer were recorded. Secondary outcomes such as the presence of postoperative wound complications, superficial peroneal or sural nerve neuritis, development of incisional neuromas, and complex regional pain syndrome were recorded. The recurrence of ankle instability after surgical treatment requiring revision was noted.

All patients underwent Brostrom-Gould repair using the inclusion of the inferior extensor retinaculum to augment the native ATFL repair, as first described by Gould et al.^11^ Adjuvant procedures were individualized to each patient. All patients were kept nonweight bearing for 4 to 6 weeks, depending on their comorbidities, followed by a progression from partial to full weight-bearing (adj) using a post-op boot and ankle brace. Patients had a clinical follow-up at 2 weeks, 6 weeks, 12 weeks, 6 months, and 1 year postoperatively.

Patients were asked to complete a postoperative survey that included the FAAM, Brief Resilience Scale (BRS), and two PROMIS domains including: Physical function (PF) and Pain Interference (PI). Patients who were unable to complete the survey in person were contacted via phone. Completion of the patient-reported outcomes survey was required for inclusion in this study. Of the 173 eligible patients, we received a total of 89 responses (94 feet) in 89 patients, as five patients had bilateral operations.

### Patient-Reported Outcome Measurement Information System: Pain Interference and Physical Function

PROMIS PI and PF domains were created using modern item response theory as a collaborative effort spearheaded by the National Institutes of Health. These instruments were designed to enable quantification and stratification of patient health states across a wide spectrum of underlying variables. Computerized adaptive algorithms enable the instruments to be customized to each individual participant based on their previous responses. The algorithm customizes each survey by drawing from a large array of questions for each underlying domain (121 questions for PF and 40 for PI).^28^ PROMIS scores are reported in a standardized fashion because a score of 50 represents the general population average with a standard deviation of 10. The interpretation of PROMIS scores is based on the connotation associated with the characteristic being measured. As an example, a low PI score (less pain) would be viewed positively, whereas a low PF score (more disability) would be viewed as a negative outcome.^28^

### Foot Ankle Ability Measure

The FAAM was created to assess changes in patient-reported PF for individuals with foot and ankle disorders in 2005.^21^ The instrument consists of a 21-question subscale designed to assess activities of daily living (ADL) and an eight questions sport subscale (Supplemental File 1, http://links.lww.com/JG9/A171). All questions are scored on a 4-point scale and then transformed into a percentage to yield a final value. For both subscales, a higher percentage equates to a higher level of physical capacity. Multiple studies have shown the FAAM to be valid, reliable, and potentially a preferred instrument for evaluation of patients with CAI.^20,21,22,23,24,25^

### Brief Resilience Scale

The BRS was originally created in 2008 and aimed to capture the true essence of resilience that the authors defined as “the ability to bounce back from stress.”^29^ The scale is based on a series of six questions scored on a five-point Likert scale with three of the six questions scored in reverse (Supplemental File 2, http://links.lww.com/JG9/A172). The total score is then averaged to yield the final BRS value, with higher averages indicating increased resilience. The BRS has been shown to possess excellent psychometric properties.^1^ It has also been used in high-demand populations such as athletes and the active duty military population.^30,31^ The population means of BRS scores for patients undergoing orthopaedic surgery are currently unknown.^5^

### Statistical Analysis

Data was aggregated in Microsoft Excel and entered in SPSS v. 27 for statistical analysis. All continuous variables were first evaluated for normality via the use of a Shapiro-Wilk test. After checking for normality, Mann-Whitney *U* tests and Spearman correlations were used to assess the association of categorical and continuous variables, respectively, on PROMIS and FAAM domains. A linear regression model was constructed to evaluate the independent effect of resilience on each PROMIS and FAAM outcome instrument. Variables were included in the regression model based on an a priori significance threshold of *P* < 0.05 in bivariate analysis.

## Results

### Clinical Outcomes

Most the cohort were women (68.5%, 61/89), with a median age of 45.50 and an interquartile range (IQR) of 20 years (Table 1). The median body mass index was 31.1 with an IQR of 13.5. Eighteen patients (20.2%) had undergone previous surgical procedures for the treatment of CAI, whereas 10 patients (11.2%) had a history of major trauma as the primary cause of CAI. A total of 11 patients (12.4%) reported engaging in athletic activities before surgery. The prevalence of patient comorbidities is presented in Table 1. Approximately 95% of patients (85/89) had CAI in the presence of an associated copathology. The prevalence of specific associated copathologies is also presented in Table 1. As a result, 80 of 89 (89.9%) patients also underwent an adjuvant procedure in addition to open Brostrom-Gould repair (Table 1). Complications were observed in 5 (5.5%) patients with one of the two patients who experienced wound complications requiring an surgical incision and drainage. One patient developed complex regional pain syndrome within the first 6 months after the procedure. A total of four patients (4.5%) experienced recurrence of lateral ankle instability at a median of 8 months postoperatively. Three of the four patients who experienced recurrence had previously undergone procedures for ankle instability (*P* = 0.025). All of these patients underwent revision lateral ligament reconstructions without further recurrence of lateral ankle instability.

**Table 1:** Cohort Demographics, Procedural Characteristics, and Clinical Outcomes

Characteristics	Median (IQR) or N (%)
Age	45.0 (20)
BMI	31.1 (13.5)
Sex	
Male	28 (31.5)
Female	61 (68.5)
Race	
White	66 (74.2)
African American & Hispanic	23 (25.8)
Previous ankle instability operation	18 (20.2)
History of major trauma	10 (11.2)
Athlete	11 (12.4)
Comorbidities and substance use	
Smoking	12 (13.5)
Diabetes	11 (12.4)
Rheumatoid arthritis	2 (2.2)
Collagen pathologies	2 (2.2)
Generalized hyperlaxity	5 (5.6)
Positive anterior drawer test	43 (48.3)
Concomitant pathologies	
Talar osteochondral defect	16 (18.0)
Tibial osteochondral defect	3 (3.4)
Peroneal tendinopathy	73 (82.0)
Deltoid ligament injury	2 (2.2)
Talar tilt varus	22 (24.7)
Preoperative ankle osteoarthritis	12 (13.5)
Anterior impingement	6 (6.7)
Posterior impingement	25 (28.1)
Adjuvant operations performed	
Ankle arthroscopy	27 (30.3)
Calcaneal osteotomy	21 (23.6)
Peroneal débridement	73 (82.0)
Peroneal groove deepening	4 (4.5)
Peroneal tenodesis	18 (20.2)
Fibularis longus to brevis transfer	9 (10.1)
Complications	
All complications	6 (6.7)
Wound complication	2 (2.2)
Superficial peroneal nerve injury	0 (0)
Sural nerve injury	3 (3.4)
Incisional neuroma	0 (0)
Reflex sympathetic dystrophy	1 (1.1)
Reoperations	2 (2.2)
Recurrence of instability	
Failure	4 (4.5)
Time to failure	8.5 months (14)
Revision lateral ligament procedures	4 (4.5)

BMI = body mass index, IQR = interquartile range

### Patient Reported Outcomes

Patient-reported outcomes instruments were collected at a median of 4.0 years postoperatively with an IQR of 5.2 (Table 2). The median PROMIS PF and PI scores were 45.7 (10.6 IQR) and 52.6 (16.5 IQR), respectively. However, the median FAAM activity of daily living and sport subscales were 76.1 (35.6 IQR) and 32.1 (74.1 IQR), respectively. The average BRS score was 4.1, with a SD of 0.5. PROMIS PF and PI domains correlated strongly with both of the FAAM subscales (|r >0.598|, *P* < 0.001 for all).

**Table 2:** Patient-Reported Outcomes of Brostrom-Gould

Patient Reported Outcome	Median Scores (IQR)
Time from surgery to survey (yr)	4.0 (5.2)
PROMIS domains	
PROMIS physical function	45.7 (10.6)
PROMIS pain interference	52.6 (16.5)
FAAM scores	
FAAM ADL	76.1 (35.6)
FAAM S	32.1 (74.1)

ADL = activities of daily living, FAAM = Foot Ankle Ability Measure, IQR = interquartile range, PROMIS, Patient-Reported Outcome Measurement Information System

### Bivariate Analysis

The results of the exploratory bivariate analysis for the factors affecting patient-reported outcomes are shown in Supplemental Table 1, http://links.lww.com/JG9/A170. Notably, resilience was found to have a moderate correlation with PROMIS PI (r = −0.378, *P ≤* 0.001). Resilience exhibited a strong relationship with PROMIS PF (r = 0.420, *P* < 0.001), FAAM activity of daily living subscale (r = 0.424, *P* < 0 .001), and the FAAM sport subscale (r = 0.401, *P* < 0.001). Patients who reported engaging in athletic activity had significantly higher levels of function and less pain across all outcome's measures (*P* < 0.011 for all, Supplemental Table 1, http://links.lww.com/JG9/A170). The median PROMIS PF and PIs scores in athletes were 55.4 and 38.7, respectively, compared with 44.9 and 52.6, respectively, in nonathletes. The median FAAM ADL and sport subscale scores in athletes were 95.2 and 92.9, respectively, compared with 76.2 and 28.6 in nonathletes. In addition to resilience and athletic activity, a total of six other parameters were found to be associated or correlated with at least one outcomes variable. These variables included race, rheumatoid arthritis, peroneal tenodesis, age, body mass index, and time to patient-reported outcomes collection.

### Regression Results

Resilience was found to have an independent effect on all PROMIS and FAAM measures when controlling for the effect of confounding variables (Table 3). Resilience's effect on outcome measures was as follows: PROMIS PF (unstandardized β 8.2, 95% confidence interval [CI] 3.9 to 12.6), PROMIS PI (unstandardized β −4.8, 95% CI −7.9 to −1.7), FAAM activity of daily living subscale (unstandardized β 16.6, 95% CI 8.7 to 24.6), and FAAM sport subscale (unstandardized β 28.4, 95% CI 15.9 to 40.9). Preoperative participation in athletic activities also had a positive independent effect on multiple outcomes metrics including: PROMIS PF (unstandardized β 9.4, 95% CI 2.8 to 16.0), PROMIS PI (unstandardized β −5.3, 95% CI −10.0 to −0.582), and FAAM sport subscale scores interference (unstandardized β 34.4, 95% CI 15.4 to 53.4).

**Table 3:** Linear Regression Model for Factors Affecting Patient-Reported Outcomes

Covariates	PROMIS Physical Function β	*P*-Value
Age	−0.08	0.239
RA	−8.9	0.223
Athlete	9.4^a^	0.006^a^
Years postop at survey	0.379	0.356
Resilience	8.2^a^	<0.001^a^
	PROMIS pain interference β	
African American or Hispanic	2.0	0.272
Athlete	−5.1^a^	0.036^a^
Years postop at survey	0.285	0.181
Resilience	−4.6^a^	0.004^a^
	FAAM ADL β	
BMI	-0.259	0.330
Athlete	5.5	0.391
Years postop at survey	2.2^a^	0.005^a^
Resilience	16.6^a^	<0.001^a^
	FAAM S β	
Peroneal tenodesis	−12.8^a^	0.011^a^
Athlete	34.4^a^	0.001^a^
Years postop at survey	1.3	0.282
Resilience	28.4^a^	<0.001^a^

ADL = activities of daily living, BMI = body mass index, FAAM = Foot Ankle Ability Measure, PROMIS = Patient-Reported Outcome Measurement Information System.

a Significant value of p<.05.

## Discussion

The Brostrom-Gould procedure has become known as the benchmark surgical procedure for lateral ankle ligament repair because of a multitude of studies demonstrating favorable long-term outcomes compared with other anatomic and nonanatomic repair techniques.^14,16–18^ The clinical outcomes of this study including wound complications and recurrence of instability are all largely comparable with those in literature and add to the body of literature illuminating the satisfactory clinical results of the open modified Brostrom-Gould.^12,15,22,25,32–34^ However, little is known about the factors effecting the intermediate outcomes of the Brostrom-Gould repair as measured by new patient-reported outcomes instruments. The dearth of literature is largely because of most studies comparing outcomes in risk factors for the recurrence of lateral ankle instability. In addition, studies that have compared outcome scores between groups primarily focus on how one patient-related or treatment-driven factor affected patient-reported outcomes.^22,24,26,35^ Although studies examining the predictors of poor prognosis measured by pathology recurrence are undoubtedly of clinical value, it is important for surgeons to be made aware of patient and treatment related factors that could be affecting the functional outcomes of these patients. The principal results of this study revealed patient resilience, as measured by the BRS, and preoperative athletic status are both significant independent predictors of improved patient outcomes scores following Brostrom-Gould Repair.

Resilience is defined, most simply, as one's ability to bounce back from stress. More recently, there has been an increasing interest within the surgical community in investigating the psychologic factors that affect patient's ability to recover from surgery.^7^ Multiple studies have now highlighted the role resilience plays in the outcomes of patients undergoing orthopaedic surgery ranging from upper extremity arthroplasty to hip fracture repair and spine procedures.^4,5,7,36,37^ This is the first study to date to demonstrate the impact of resilience on the outcomes of lateral ligament repair and more generally the field of foot and ankle surgery. Tokish et al was the first to investigate the role of resilience, as measured by the BRS, within the field of orthopaedics and demonstrate its impact on patients undergoing total shoulder arthroplasty.^5^ After grouping patients based on resilience scores, they found high resilience groups to be associated with improved American Shoulder and Elbow Surgeons ASES, Single Assessment Numeric Evaluation, and Penn scores. Given the population mean for BRS scores in the orthopaedic population is unknown, artificial separation of patients into low- and high-resilience groups could confound results. Herein, we examined BRS scores (resilience) on a continuous scale while adjusting for confounders using regression analysis and found a notable effect on outcomes per unit of resilience. Importantly, resilience's effect was above the minimal clinically important difference (MCID) for all outcomes measures including: PROMIS PF (8.2 versus 4.5 MCID), PROMIS PI (−4.8 versus 4.3 MCID), FAAM ADL subscale (16.6 versus 8 MCID), and FAAM Sports Subscale (28.4 versus 13.1).^20,21^ These results demonstrate that not only were the changes associated with resilience statistically significant, but each unit increase in patient resilience was indicative of clinically significant improvements PF and decreased pain burden. Although inherently our results mean low and high resilience groups are likely to be markedly different in outcomes; our results illuminate that interventions targeting patient resilience could have a positive effect on patient outcomes without requiring major changes in underlying resilience. Outside of joint arthroplasty, Coronado et al found that resilience had an independent effect on patient's 12 month PF, pain interreference, social participation, and disability scores in patients undergoing laminectomy with and without fusion.^37^ Compared with our results, the previous study found a slightly smaller correlation of resilience with PROMIS PF (r = 0.37 versus 0.43) and larger correlation of resilience with PROMIS PI (r = −0.41 versus −0.38). These results are largely comparable despite extremely different patient populations and surgical interventions. Furthermore, high resilience groups have also been shown to have better early postoperative outcomes and return to duty in military personnel undergoing arthroscopic knee procedures.^31^ Drayer et al found, in this previous study, a correlation between resilience and PROMIS PI of r = −0.19, significantly weaker than the correlation found in our study of r = −0.38. This difference could potentially be attributed to Drayer et al using a static version of the PROMIS instruments, which might not as accurately detect changes in pain levels when compared with the computerized adaptive PROMIS instruments used in our study and that of Coronado et al. In this context, our results add to the growing evidence supporting the generalizability of resilience and its impact on the outcomes of surgical intervention across a wide variety of orthopaedic procedures. Preoperative quantification of patient resilience could potentially allow clinicians to better prognosticate patients undergoing foot and ankle surgery and more specifically lateral ankle ligament repair. Surgeons should be increasingly aware of the impact psychosocial factors, such as resilience, play in postoperative patient outcomes, and work to incorporate methods to quantify these factors into standard practice.

The high prevalence of lateral ankle instability in the athletic population has resulted in most studies investigating the outcomes of open Brostrom-Gould consisting of primarily athletically active patients.^14,15,22,25,38^ Interestingly, no study to date has evaluated the effect of preoperative athletic activity on surgical outcomes compared with more sedentary individuals. Cho et al reported a mean FAAM ADL and Sports subscale scores of 95.2 and 89 1 respectively at a minimum follow up of 2 years in a randomized prospective cohort study with a modified-Brostrom group.^15^ In a retrospective review of 81 Modified Brostrom-Gould repairs with suture tape augmentation, Coetzee et al^25^ found median FAAM ADL and sport subscale scores of 97.6 and 93.8 at a minimum of 6 months postoperative follow-up. Compared with our study, both previous studies reported markedly higher FAAM outcome scores at the final follow-up. In these studies, the percentage of patients who engaged in athletic activities was significantly higher at 100% & 90%, respectively, compared with 12% in this study. The effect of patient's athletic status is a potential rationale for the lower FAAM scores seen in our cohort. Interestingly, Buerer et al reported a lower mean FAAM ADL and sports subscale scores of 82.5 and 82.2 in a cohort with only 58% of patients reporting engaging in athletic activity. Our results showed median FAAM ADL and sports subscale scores (95.2 and 92.9, respectively) for athletes that were comparable with these previous outcomes. In addition, we found patient's preoperative athletic status had an independent effect above the MCID for PROMIS PF (9.4 versus 4.5 MCID), PI (−4.6 versus 4.3 MCID), and FAAM sport subscale (34.4 versus 13.1).^20,21^ Although the explanation of the effect of athletic participation on outcomes is likely multifactorial and could be a result of higher levels of preoperative function, our results showed the clinically notable independent effect of athletic status on the intermediate term outcomes of the modified Brostrom-Gould Procedure. Surgeons should be aware of this dichotomy when prognosticating patients undergoing lateral ligament reconstruction.

Although the patient's athletic status is an unmodifiable factor affecting outcomes of the Brostrom-Gould, patient resilience may be a modifiable factor that allows surgeons to improve patient's postoperative recovery. Interventions targeting patient resilience have been investigated, but no study to date has studied a resilience intervention in patients undergoing surgical procedures.^39^ Preliminary signs of efficacy have been observed in some trials using methods such as cognitive behavioral therapy and mindfulness training.^39^ The strongest effect on resilience was noted when these techniques were used in combination.^39^ Notably, McGonagle et al^40^ found psychotherapy “coaching” sessions conducted over the telephone could improve patient resilience. This previous study demonstrated interventions targeting resilience could be conducted with greater patient convenience and less burden on the health system. Further studies investigating presurgical and postsurgical resilience interventions are warranted to better understand how surgeons can improve the outcomes of all patients.

The first limitation of this study is because of its retrospective nature and, therefore, has the potential to be subject to multiple biases known to afflict this design. Second, we lacked 100% follow-up of patient-reported outcomes for all potential study participants, and this may have affected our results. Third, our study also lacked preoperative PROMIS and FAAM scores limiting our ability to assess the amount of improvement seen in these metrics postoperatively, assess baseline levels of function, or evaluate any potential interconnection of these baseline levels with resilience. Fourth, we encourage readers to interpret our findings regarding the positive effect of athletic activity on functional outcomes with caution, as athletes comprised only 12% of this study population. Larger studies comparing the outcomes of Brostrom-Gould procedures in athletes and non-athletes are warranted. This study also was conducted at a single institution which may limit the generalizability of our findings. Future studies could be improved by addition of more diverse geographic populations, surgical techniques, and cultural backgrounds. Last, our study lacked preoperative resilience scores, and we acknowledge that surgical intervention could affect the stability of resilience. However, Magaldi et al^36^ found resilience was “relatively stable” at three and 12 months after total knee arthroplasty compared with baseline and did not significantly change over time. In addition, Markovitz et al found resilience amongst 253 breast cancer patients undergoing treatment was not markedly different from a group of 211 healthy female controls.^41^ Taken together, these studies provide evidence to support that resilience is relatively stable following orthopaedic surgery and other stressors.^36,41^

## Conclusions

Resilience and patient participation in athletic activities are independent predictors of improved postoperative functional outcomes as measured by PROMIS and FAAM instruments at intermediate term follow-up. Resilient patients and athletes reported markedly higher PF and less pain burden postoperatively. Furthermore, preoperative quantification of resilience could enable improved prognostication of patients undergoing lateral ligament repair of the ankle. This study adds to the growing evidence supporting the importance of resilience in a patient's ability to respond to surgical stress and is the first to define this phenomenon in the orthopaedic foot and ankle population.

## Supplementary Material

SUPPLEMENTARY MATERIAL
