# Supplementary material for: Impact of Patient Resilience on Outcomes of Open Brostrom-Gould Lateral Ligament Repair
Source: J Am Acad Orthop Surg Glob Res Rev. 2021 Nov 18;5(11):e21.00103. doi: 10.5435/JAAOSGlobal-D-21-00103 (PMC8604009; doi:10.5435/JAAOSGlobal-D-21-00103)
Supplement: SUPPLEMENTARY MATERIAL [file jagrr-5-e21.00103-s001.pdf]

Foot and Ankle Ability Measure (FAAM)
Activities of Daily Living Subscale

Please Answer **<u>every question</u>** with **<u>one response</u>** that most closely describes your condition within the past week.
If the activity in question is limited by something other than your foot or ankle mark “Not Applicable” (N/A).

	No Difficulty	Slight Difficulty	Moderate Difficulty	Extreme Difficulty	Unable to do	N/A
Standing	☐	☐	☐	☐	☐	☐
Walking on even Ground	☐	☐	☐	☐	☐	☐
Walking on even ground without shoes	☐	☐	☐	☐	☐	☐
Walking up hills	☐	☐	☐	☐	☐	☐
Walking down hills	☐	☐	☐	☐	☐	☐
Going up stairs	☐	☐	☐	☐	☐	☐
Going down stairs	☐	☐	☐	☐	☐	☐
Walking on uneven ground	☐	☐	☐	☐	☐	☐
Stepping up and down curbs	☐	☐	☐	☐	☐	☐
Squatting	☐	☐	☐	☐	☐	☐
Coming up on your toes	☐	☐	☐	☐	☐	☐
Walking initially	☐	☐	☐	☐	☐	☐
Walking 5 minutes or less	☐	☐	☐	☐	☐	☐
Walking approximately 10 minutes	☐	☐	☐	☐	☐	☐
Walking 15 minutes or greater	☐	☐	☐	☐	☐	☐

Foot and Ankle Ability Measure (FAAM)
Activities of Daily Living Subscale
Page 2

Because of your foot and ankle how much difficulty do you have with:

	No Difficulty at all	Slight Difficulty	Moderate Difficulty	Extreme Difficulty	Unable to do	N/A
Home responsibilities	☐	☐	☐	☐	☐	☐
Activities of daily living	☐	☐	☐	☐	☐	☐
Personal care	☐	☐	☐	☐	☐	☐
Light to moderate work (standing, walking)	☐	☐	☐	☐	☐	☐
Heavy work (push/pulling, climbing, carrying)	☐	☐	☐	☐	☐	☐
Recreational activities	☐	☐	☐	☐	☐	☐

Martin, R; Irrgang, J; Burdett, R; Conti, S; VanSwearingen, J: Evidence of Validity for the Foot and Ankle Ability Measure. Foot and Ankle International. Vol.26, No.11: 968-983, 2005.

Foot and Ankle Ability Measure (FAAM)
Sports Subscale

Because of your foot and ankle how much difficulty do you have with:

	No Difficulty at all	Slight Difficulty	Moderate Difficulty	Extreme Difficulty	Unable to do	N/A
Running	☐	☐	☐	☐	☐	☐
Jumping	☐	☐	☐	☐	☐	☐
Landing	☐	☐	☐	☐	☐	☐
Starting and stopping quickly	☐	☐	☐	☐	☐	☐
Cutting/lateral Movements	☐	☐	☐	☐	☐	☐
Ability to perform Activity with your Normal technique	☐	☐	☐	☐	☐	☐
Ability to participate In your desired sport As long as you like	☐	☐	☐	☐	☐	☐

Martin, R; Irrgang, J; Burdett, R; Conti, S; VanSwearingen, J: Evidence of Validity for the Foot and Ankle Ability Measure. Foot and Ankle International. Vol.26, No.11: 968-983, 2005.
