# Supplementary material for: Impact of Patient Resilience on Outcomes of Open Brostrom-Gould Lateral Ligament Repair
Source: J Am Acad Orthop Surg Glob Res Rev. 2021 Nov 18;5(11):e21.00103. doi: 10.5435/JAAOSGlobal-D-21-00103 (PMC8604009; doi:10.5435/JAAOSGlobal-D-21-00103)
Supplement: SUPPLEMENTARY MATERIAL [file jagrr-5-e21.00103-s002.docx]

Brief Resilience Scale (BRS)

| Questions | Strongly Disagree | Disagree | Neutral | Agree | Strongly Agree |
| --- | --- | --- | --- | --- | --- |
| I tend to bounce back quickly after hard times | 1 | 2 | 3 | 4 | 5 |
| I have a hard time making it through stressful events. | 5 | 4 | 3 | 2 | 1 |
| It does not take me long to recover from a stressful event. | 1 | 2 | 3 | 4 | 5 |
| It is hard for me to snap back when something bad happens | 5 | 4 | 3 | 2 | 1 |
| I usually come through difficult times with little trouble. | 1 | 2 | 3 | 4 | 5 |
| I tend to take a long time to get over setbacks in my life. | 5 | 4 | 3 | 2 | 1 |

Scoring: Add the scores for all 6 items and divide by the total number of questions answered.

Smith BW, Dalen J, Wiggins K, Tooley E, Christopher P, Bernard J. The brief resilience scale: assessing the ability to bounce back. Int J Behav Med. 2008;15(3):194-200. doi: 10.1080/10705500802222972. PMID: 18696313.
