# Supplementary material for: Impact of Patient Resilience on Outcomes of Open Brostrom-Gould Lateral Ligament Repair
Source: J Am Acad Orthop Surg Glob Res Rev. 2021 Nov 18;5(11):e21.00103. doi: 10.5435/JAAOSGlobal-D-21-00103 (PMC8604009; doi:10.5435/JAAOSGlobal-D-21-00103)
Supplement: SUPPLEMENTARY MATERIAL [file jagrr-5-e21.00103-s003.pdf]

Categorical variables	PROMIS Physical Function (p value)	PROMIS Pain Interference (p value)	FFI Pain (p value)	FFI Disability (p value)
**Sex**	.649	.714	.085	.677
**Race**	.111	**.027***	.086	.282
**Prior ankle instability operation**	.927	.733	.660	.482
**History of major trauma**	.554	.515	.649	.680
**Athlete**	**.001***	**.004***	**.011***	**.001***
**Comorbidities and substance use**				
**Smoking**	.239	.164	.256	.152
**Diabetes**	.769	.492	.930	.854
**RA**	**.033***	.115	.086	.775
**Collagen pathologies**	.080	.838	.332	.335
**Generalized Hyperlaxity**	.675	.928	.521	.515
**Anterior Drawer Positive**	.739	.712	.818	.545
**Concomitant Pathologies**				
**Talar OCD**	.143	.513	.269	.127
**Tibial OCD**	.425	.098	.228	.109
**Peroneal Tendinopathy**	.979	.376	.712	.550
**Deltoid ligament injury**	1.000	.944	.680	.320
**Talar Tilt Varus**	.353	.096	.102	.284
**Preoperative ankle osteoarthritis**	.727	.658	.552	.950
**Anterior impingement**	.126	.403	.235	.527
**Posterior Impingement**	.502	.209	.490	.662
**Adjuvant Operations Performed**				
**Ankle arthroscopy**	.469	.839	.936	.624
**Calcaneal Osteotomy**	.527	.120	.122	.376
**Peroneal Debridement**	.646	.623	.902	.946
**Peroneal Groove Deepening**	.681	.626	.979	.936
**Peroneal Tenodesis**	.709	.374	.736	**.037***
**Fibularis Longus to Brevis Transfer**	.826	.584	.619	.994
**Complications**				
**All complications**	.084	.286	.097	.119
**Wound complication**	.484	.439	.415	.175
**Sural Nerve Injury**	.179	.873	.537	.597
**Reflex Sympathetic Dystrophy**	.831	.427	.090	.425
**Reoperations**	.207	.408	.239	.182
**Recurrence of instability**				
**Failure**	.163	.128	.266	.570
**Continuous variables**	**PROMIS Physical Function R value (p value)**	**PROMIS Pain Interference R value (p value)**	**FAAM ADL R value (p value)**	**FAAM Sports R value (p value)**
**Age**	**-.224 (p=.035*)**	-.178 (p=.096)	-.160 (p=.135)	-.134 (p=.237)
**BMI**	-.184 (p=.085)	.095 (p=.374)	**-.210 (p=.048)***	-.103 (p=.364)
**Time to Survey**	**.255 (p=.016)***	**-.246 (p=.020)***	**.337 (p=.001)***	**.247 (p=.027)***
**Resilience**	**.430 (p<.001)***	**-.378 (p<.001)***	**.424 (p<.001)***	**.401 (p=.001)***
